# Fibroblast Growth Factors in Depression

**DOI:** 10.3389/fphar.2019.00060

**Published:** 2019-02-05

**Authors:** Zheng Deng, Sheng Deng, Mu-Rong Zhang, Mi-Mi Tang

**Affiliations:** ^1^Hospital Evaluation Office, Xiangya Hospital, Central South University, Changsha, China; ^2^Department of Pharmacy, Xiangya Hospital, Central South University, Changsha, China; ^3^Institute of Hospital Pharmacy, Central South University, Changsha, China; ^4^Xiangya School of Pharmaceutical Sciences, Central South University, Changsha, China

**Keywords:** major depressive disorder, depression, fibroblast growth factor, fibroblast growth factor receptor, hippocampus

## Abstract

Major depressive disorder (MDD) is one of the most serious diseases and now becomes a major public health problem in the world. The pathogenesis of depression remains poorly understood. Fibroblast growth factors (FGFs) belong to a large family of growth factors that are involved in brain development during early periods as well as maintenance and repair throughout adulthood. In recent years, studies have found a correlation between the members of the FGF system and depression. These signaling molecules may be expected to be biomarkers for the diagnosis and prognosis of MDD, and may provide new drug targets for the treatment of depression. Here, we reviewed the correlation between some members of the FGF system and depression.

Major depressive disorder (MDD), a common chronic mental disorder, is mainly manifested by changes in emotion, cognition, behavior, sleep and appetite, and it can result in impaired overall social function. MDD is among the leading causes of suicide and disability. The prevalence of MDD is high, with nearly one in four women or six men suffering from MDD (Kessler et al., 2010). In addition, relapse is common in MDD. Approximately 60% of first-onset depressed patients will suffer from a second episode (Monroe and Harkness, 2011). In sharp contrast to high prevalence rate, the recognition rate of MDD is quite low. Among the existing MDD patients in China, only a few have received relevant treatment, and a majority of patients can’t be diagnosed and treated effectively and timely. The pathogenesis of MDD is very complex, and it has not been uniformly determined. Fibroblast growth factors (FGFs) belong to a large family of growth factors that are involved in brain development during early periods as well as maintenance and repair throughout adulthood (Terwisscha van Scheltinga et al., 2013). Many studies show that some members of FGF system are correlated with depression. We used “fibroblast growth factor” or “FGF” and mood related words (e.g., “major depressive disorder,” “depression,” “mood disorder,” or “stress”) to perform electronic searches of the English-language literatures in PubMed. References of the retrieved articles were manually screened to identify other relevant publications. Here, we mainly concentrated on the reported depression-related FGF system members, including FGFR1, FGF2, FGF9, FGF21, and FGF22.

## Fibroblast Growth Factors and Fibroblast Growth Factor Receptors

The FGF system is composed of twenty-two ligands and five receptors at present in humans and is distributed throughout the central nervous system (Turner et al., 2006). FGFs play a key role in the function, development and metabolism of neuron. During development, FGFs control the growth and morphology of several brain structures, and in the later stages of life, they continue to regulate neurogenesis, axon growth, neuroprotection, and nervous tissue maintenance and repair (Terwisscha van Scheltinga et al., 2010). Although structurally related, FGFs show different action patterns, secretory mechanisms and biological effects. Thereby they are divided into several subfamilies (Table 1), and each subfamily has its genetic and functional similarity (Itoh and Ornitz, 2004; Goldfarb, 2005; Beenken and Mohammadi, 2009; Hui et al., 2018). Unlike other subfamilies, FGF homologous factor (FHF) subfamily cannot activate FGF receptors (Olsen et al., 2003).

**Table 1:** FGF subfamilies and their members (Itoh and Ornitz, 2004; Goldfarb, 2005; Beenken and Mohammadi, 2009; Hui et al., 2018).

FGF subfamily	Members	Binding receptor	Potential effects	Secretory patterns
FGF1 subfamily	FGF1	all FGF receptors	FGF1 subfamily members have some therapeutic potential for cardiovascular disorders, mood disorders, cancers, and are widely used for wound healing.	Paracrine Subfamilies (Cofactor: heparin)
	FGF2	FGFR1c,3c,2c,1b,4		
FGF4 subfamily	FGF4, FGF5, FGF6	FGFR1c,2c,3c,4	FGF4 subfamily members have wide-ranging functions in development, including cardiac valve leaflet formation and limb development.	
FGF7 subfamily	FGF3, FGF7, FGF10, FGF22	FGFR2b,1b	FGF7 and FGF10, also known as keratinocyte growth factors, act as presynaptic organizers with roles in vesicle clustering and neurite branching. Recombinant FGF7 is widely used for wound healing.	
FGF8 subfamily	FGF8, FGF17, FGF18	FGFR3c,4,2c,1c,3b	FGF8 is involved in brain, limb, ear and eye development.	
FGF9 subfamily	FGF9, FGF16, FGF20	FGFR3c,2c,1c,3b,4	FGF20 is a neurotrophic factor for rat midbrain dopaminergic neurons, and may show promise in stem cell biology.	
FGF19 subfamily	FGF19, FGF21, FGF23	FGFR1c,2c,3c,4 (Weak activity)	Since Klotho expression is tissue-specific, the functions of FGF19 subfamily members are mainly related to human metabolic processes, such as regulating the balance of bile acid, cholesterol, glucose, vitamin D, and phosphate.	Endocrine Subfamily (Cofactor: Klotho)
FGF homologous factor (FHF) subfamily	FGF11 (FHF3), FGF12 (FHF1), FGF13 (FHF2), FGF14 (FHF4)	FHFs have high sequence identity with other FGF family members, but do not activate FGFR. FHFs bind to intracellular domains of voltage-gated sodium channels (VGSCs) and to a neuronal MAPK scaffold protein.	FHF deficits induce neurological syndromes, such as impaired central nervous system function.	/

*FGF, fibroblast growth factor; FGFR, fibroblast growth factor receptor; FHF, FGF homologous factor; MAPK, mitogen-activated protein kinase; VGSCs, voltage-gated sodium channels*.

Fibroblast growth factor receptor (FGFR), a member of the receptor tyrosine kinase family, includes five kinds of receptors, among which FGFR1∼FGFR4 have receptor activity while FGFR5 doesn’t due to the absence of intracellular part (Yeh et al., 2003; Itoh and Ornitz, 2011). Each receptor has a unique affinity for FGFs, and this uniqueness depends on the differential splicing of the third extracellular immunoglobulin-like domain (Ig-III) of the receptor variant (Viollet and Doherty, 1997).

Interactions of FGFs with their receptors are modulated by cofactors such as heparan sulfate (HS) and Klotho (Ornitz and Itoh, 2015; Hui et al., 2018). After FGFs bind to FGFRs, four key signaling pathways, including RAS-RAF-MAPK, PI3K-AKT, STAT, and PLCγ, are activated to influence gene transcription (Itoh and Ornitz, 2011; Turner et al., 2012).

## FGF Receptors and Depression

### FGFR1

Fibroblast growth factor receptor 1 (FGFR1) is widely distributed throughout the nervous system, contributing to hippocampal nerve growth and long-term potentiation (Zhao et al., 2007; Terwisscha van Scheltinga et al., 2010). The expression of FGFR1 is high in CA2 and CA3 regions of hippocampus, but low in cortex. One study shows that the wide expression of FGFR1 in embryonic neural progenitor cells plays a role in the formation of the cortical plate and the structure of gyrus and sulcus (Shin et al., 2004).

Turner C.A. et al. found that the mRNA expression of FGFR1 was down-regulated in the hippocampus of acute social defeat rats (Turner et al., 2008a). FGF2 microinjection (200 ng) into the lateral ventricle of rats, which exerts antidepressant-like effects, up-regulates the FGFR1 mRNA in the dentate gyrus (Turner et al., 2008b). Later, Aurbach E.L. et al. found that slow administration of exogenous FGF9 increased anxiety and depression-like behaviors, and decreased the expression of FGFR1 in the dentate gyrus (Aurbach et al., 2015). These changes in FGFR1 caused by the administration of exogenous FGF2/9 may result from an increase in FGF2/9 in animals or changes in depression status. These findings provide evidence for the potential therapeutic value of FGFR1 in depression.

Although an early microassay study found that the FGFR1 expression did not change in the dorsolateral prefrontal lobe and anterior cingulate gyrus of depressed subjects, a subsequent study found that FGFR1 mRNA was increased in hippocampal CA1, CA4 and dentate nuclei zone of non-psychotic depressed patients compared with healthy controls (Evans et al., 2004; Gaughran et al., 2006). Also, a significant increase in FGFR1 gene expression was found in the prefrontal cortex of postmortem depressed patients (Tochigi et al., 2008; Goswami et al., 2013). The difference in these results may be due to the factors such as sample pH, postmortem interval. Variation in anatomical distribution of FGFR1 and differences in sample size and detection methods may also affect the results.

Furthermore, FGFR1 can interact with other type of receptors to play a role in depression. FGFR1–5-HT1A (5-hydroxytryptamine receptor 1A) heteroreceptor complexes were first observed in 2012, and combined FGF2 and 5-HT1A agonist treatment increased the formation of the complexes and enhanced the synergistic receptor-receptor interactions, which was associated with the development of antidepressant effects (Borroto-Escuela et al., 2012; Borroto-Escuela et al., 2016). The results of these studies may combine the serotonin hypothesis and the neurotrophic factor hypothesis of depression together.

### FGFR2 and FGFR3

Fibroblast growth factor receptor 2 (FGFR2) and fibroblast growth factor receptor 3 (FGFR3) are weakly expressed in astrocytes of the hippocampus. The expression level of FGFR2 increases with age in the adult stage, while the expression level of FGFR3 remains relatively unchanged in the early development of the adult stage (Bansal et al., 2003). FGFR2 is mainly expressed in diencephalon, telencephalon and mesencephalon, and plays an important role in neurogenesis, neuroprotection, nerve repair and hippocampal learning (Alzheimer and Werner, 2002; Umemori et al., 2004; Stevens et al., 2012), while FGFR3 is mainly expressed in diencephalic glial cells, and plays a role in the development of the caudal telencephalon (Ford-Perriss et al., 2001; Moldrich et al., 2011).

No change in the expression of FGFR2, FGFR3, and FGFR4 was found in the prefrontal cortex of postmortem depressed patients (Goswami et al., 2013). However, another study found decreased expression of FGFR2 and FGFR3 in the dorsolateral prefrontal cortex (DLPFC) and the anterior cingulate cortex (AnCg) after autopsy on patients with depression (Evans et al., 2004). Decreased expression of FGFR2 and FGFR3 was also found in the hippocampus of postmortem depressed patients (Aurbach et al., 2015).

## FGFs and Depression

Currently, among the twenty-two identified FGFs, FGF2, FGF9, FGF21, and FGF22 have been found to be associated with depression.

### FGF2

FGF2, widely expressed throughout the central nervous system, is one of the main neurotrophic factors. FGF2 can regulate hippocampal neurogenesis, synaptic formation and growth, and thereby affects learning, memory, long-term potentiation and response to injury (Flores et al., 2002; Ganat et al., 2002; Rai et al., 2007). FGF2 is mainly expressed in neurons and glial cells during puberty and adulthood, and the hippocampus expresses the highest level of FGF2 and its receptors in the brain (Ford-Perriss et al., 2001).

Several experiments demonstrated that the mRNA expression of FGF2 was down-regulated in acute social defeat rats and post-stroke depressed rats (Turner et al., 2008a; Ji et al., 2014). Interestingly, FGF2 levels were up-regulated after antidepressant administration. Maragnoli et al. found that FGF2 mRNA levels in the prefrontal cortex of rats were up-regulated after short-term or long-term combination of fluoxetine and olanzapine (Maragnoli et al., 2004). Similarly, after continuous injection of desipramine or fluoxetine on adult rats for 2 weeks, immunoreactivity of FGF2 was increased in neurons of the cerebral cortex and in astrocytes of the hippocampus (Bachis et al., 2008). One of the underlying mechanisms that how antidepressants up-regulate the levels of FGF2 involves extracellular signal-regulated kinase (ERK)-dependent early growth response 1 (EGR1) signaling pathway in astrocytes. Amitriptyline may activate FGFR and epidermal growth factor receptor (EGFR), which induces the phosphorylation of ERK1/2. Then it increases the expression of EGR1, which in turn, increases FGF2 expression (Kajitani et al., 2015). A number of animal studies have placed their focus on the effects of FGF2 administration. FGF2 microinjection into the lateral ventricle of depressed rats was reported to have antidepressant effects (Turner et al., 2008b). Moreover, FGF2 treatment could diminish neuronal death in olfactory bulbectomy (OBX) rats, improve OBX induced reduction in hippocampal neurogenesis and reverse OBX induced depression-like behavior (Jarosik et al., 2011). Furthermore, the researchers found that FGF2, rather than amitriptyline, could reverse the behavior alterations in FGF2 knockout mice, indicating FGF2 as a mediator in the antidepressant effects of amitriptyline. Similarly, slow infusion of FGF2 showed antidepressant effects in chronic unpredictable stress (CUS) rats, and the treatment of antidepressants failed to improve depression-like behaviors after the administration of FGFR inhibitors (Elsayed et al., 2012). Though the pathogenesis of depression and how FGFs exert antidepressant effects are unclear, researchers still get some clues from experiments. In chronic unpredictable mild stress (CUMS) mice, FGF2 increased the phosphorylation of ERK and AKT via binding to FGFR1, which up-regulated the expression of Bcl-2, leading to decreased caspase-3 levels. These changes mediated neuroprotective effects and in turn exerted antidepressant effects (Wang et al., 2018). By activating ERK/MAPK pathway, FGF2 could also increase the expression of glucocorticoid receptor and might reduce the likelihood of depression (Numakawa et al., 2018). FGF2-ERK signaling pathway was also reported to be involved in the neuroinflammation induced model of depression. In this model, exogenous FGF2 alleviated inflammation-induced impairment in hippocampal neurogenesis by phosphorylating ERK1/2, and reversed depressive-like behavior (Tang et al., 2017). Meanwhile, exogenous FGF2 could modulate depressive-like behavior by affecting the levels of pro-inflammatory cytokines (including interlukin-1β, interlukin-6, and tumor necrosis factor-α), anti-inflammatory cytokine (interlukin-10) and CX3CL1 (Tang et al., 2018). In brief, the antidepressant effects of FGF2 may be mediated by several signaling pathways and then achieved by neuronal remodeling (including neurogenesis, alterations of synaptic connections and plasticity) and regulation of monoamine neurotransmitter system.

Postmortem studies concerning FGF2 levels in depressive patients were inconsistent. Evans et al. found that the levels of FGF2 in the DLPFC and the AnCg of postmortem depressed patients were significantly lower than those of healthy controls (Evans et al., 2004). Gaughran F. et al. observed decreased FGF2 mRNA in the hippocampal CA4 region of postmortem non-psychotic major depressive patients (Gaughran et al., 2006). However, no change in FGF2 gene expression was found in the prefrontal cortex of postmortem depressive patients (Goswami et al., 2013). Further studies are needed to discover the changes of FGF2 in different brain regions.

There were discrepant results of clinical studies on the relationship between FGF2 and depression. In an early report, no significant difference in plasma FGF2 levels was found between depressive patients and healthy controls (Takebayashi et al., 2010). But in this study, patients with MDD were under remission by being treated with antidepressants or mood stabilizers. In another study, decreased serum FGF2 levels were found in depressive patients, and serum FGF2 levels decreased significantly but marginally after 8 weeks of treatment (He et al., 2014). In contrast, Kahl et al. found that serum FGF2 concentrations were increased in depressed patients with borderline personality disorder (Kahl et al., 2009). A similar result was found that the FGF2 levels in the peripheral blood of MDD patients with childhood trauma exposure were significantly higher than those of healthy controls (Lu et al., 2013). In a latest meta-analysis, peripheral FGF2 levels of depressive patients were significantly higher than those of healthy controls, while no significant difference of FGF2 levels in the central nervous system was found (Wu et al., 2016). One of the reasons for the inconsistent in these results is that some depressed patients had been treated when they were enrolled in studies, while other patients were drug free. Another reason is that some patients had comorbidities or special personal histories other than MDD. Different observation time or different ages may also affect the results.

### FGF9

FGF9, mainly expressed by neurons in the cortex, hippocampus, thalamus, cerebellum and spinal cord, can promote cell survival and inhibit the differentiation of astrocytes (Tagashira et al., 1995; Nakamura et al., 1997; Todo et al., 1998; Garces et al., 2000; Kanda et al., 2000; Huang et al., 2009; Lin et al., 2009; Lum et al., 2009). FGF9 not only activates IIIc splice variant of FGFR3, but also activates IIIb splice variant of FGFR3. This binding specificity makes FGF9 relatively special to other subfamily members (Ornitz et al., 1996; Santos-Ocampo et al., 1996).

The mRNA expression of FGF9 in the CA1, CA2, CA3 and dentate gyrus of hippocampus was up-regulated in social defeat stress rats (Aurbach et al., 2015). Further experiments found that administrating exogenous FGF9 increased anxiety- and depression-like behaviors, while knocking down endogenous FGF9 expression in the dentate gyrus by lentiviral vector showed decreased anxiety-like behavior in rats (Aurbach et al., 2015). It is well to be reminded that, FGF2 and FGF9 were found to have the opposite effect through chronic administration of FGFs, that is, FGF2 reduced anxiety- and depression-like behaviors, while FGF9 increased anxiety- and depression-like behaviors (Turner et al., 2008b; Perez et al., 2009). These animal studies indicate that FGF9 has an anxiogenic and prodepressant role in the rodent brain. The balance between FGF factors may be crucial to emotional regulation.

Several human studies provide evidence that FGF9 expression was increased in certain brain areas of depressed patients, such as hippocampus, frontal cortex and locus coeruleus (Evans et al., 2004; Bernard et al., 2011; Aurbach et al., 2015). Another study found no change in the expression of FGF9 gene in the prefrontal cortex of postmortem depressive patients (Goswami et al., 2013). These results suggest that high levels of FGF9 in the brain may exert an important role in the development of mood disorders.

### FGF21

FGF21, an important endogenous regulator of glucose and lipid metabolism, has been proposed as a therapeutic target for diabetes and obesity (Inagaki et al., 2007). It also has a strong neuroprotective effect and acts as a mediator of some mood stabilizers (Leng et al., 2015). Liver is the main organ that produces FGF21 (Kharitonenkov and Larsen, 2011). Peripheral FGF21 could reach the brain and play a beneficial role in central nervous system (Sarruf et al., 2010). FGF21 can also be detected in human cerebrospinal fluid (Tan et al., 2011, 2013). FGF21 contains 181 amino acid residues and has a molecular weight of about 20 kDa, which is derived from the mature protein of 209 amino acid residues encoded by the FGF21 gene on chromosome 19 (Nishimura et al., 2000).

In a recent study, the FGF21 levels in cerebrospinal fluid were found negatively related to the score of Beck Depression Inventory (BDI) in male subjects. The lower the FGF21 levels were, the more severe the depression was. However, no significant correlation was found in female subjects (Liu et al., 2017). The gender difference of the results may be related to estrogen or oxytocin.

### FGF22

As a member of FGF family, FGF22 has been found to be significantly associated with the occurrence of epilepsy, cancer, depression and other diseases as well as embryonic brain development (Jarosz et al., 2012; Singh et al., 2012; Lee and Umemori, 2013; Miyake and Itoh, 2013). An animal experiment confirmed that FGF22 was involved in the formation of excitatory synapses in hippocampal neurons (Williams et al., 2016). FGF22 is mainly expressed in the brain and skin, and its receptor is FGFR2 (Nakatake et al., 2001; Evans et al., 2004; Beenken and Mohammadi, 2009).

FGF22 knockout mice models display depression-like behaviors (including despair and anhedonia) in forced swim test, tail suspension test and sucrose preference test (Williams et al., 2016). The loss of excitatory synapses in the hippocampus induced by the absence of FGF22 and FGFR2 interaction may partly explain the depressive behavior of FGF22 knockout mice.

In a controlled study of 90 first-onset depressive patients and 90 healthy volunteers, decreased serum FGF22 levels were found in depressive patients, and these levels were negatively correlated with the patients’ scores of Hamilton Depression Scale. After treatment, FGF22 levels increased along with the relief of depressive symptoms (Xu et al., 2017).

### Other FGFs

Evans et al. performed autopsies on depressive patients, and found decreased expression of FGF1 in the DLPFC and AnCg, and increased expression of FGF12 in the AnCg (Evans et al., 2004). Major information of postmortem studies and clinical researches included in this review is listed in Tables 2 and 3, respectively.

**Table 2:** Postmortem studies concerning the correlation between FGFs and depression.

Study	Samples	Comparison	Numbers	FGF	Main results
Evans et al. (2004)	DLPFC, AnCg	MDD	4	FGF2	FGF2 levels were lower in the AnCg of MDD patients.
		HC	6	FGF9	FGF9 expression in the DLPFC was increased in MDD patients.
				FGF1	FGF1 levels were decreased in the DLPFC and AnCg of depressive patients.
				FGF12	Increased FGF12 was observed in the AnCg of depressive patients.
Gaughran et al. (2006)	Hippocampus	MDD	9	FGF2	Decreased FGF2 mRNA was found in the hippocampal CA4 region.
		HC	12		
Goswami et al. (2013)	Prefrontal cortex	MDD	16	FGF2	No change in FGF2 gene expression was found in the prefrontal cortex of postmortem depressive patients.
		HC	16		
Bernard et al. (2011)	locus coeruleus	MDD	12	FGF9	FGF9 expression was increased in depressed patients.
		HC	8		
Goswami et al. (2013)	Prefrontal cortex	MDD	16	FGF9	No change was found in the expression of FGF9 gene in the prefrontal cortex of postmortem depressive patients.
		HC	16		
Aurbach et al. (2015)	Hippocampus	MDD	36	FGF9	FGF9 expression was increased in depressed patients.
		HC	56		

*AnCg, anterior cingulate cortex; DLPFC, dorsolateral prefrontal cortex; FGF, fibroblast growth factor; HC, healthy control; MDD, major depressive disorder*.

**Table 3:** Main information of clinical researches studying the correlation between FGFs and depression.

Study	Samples	Depression scale used	Comorbidity with anxiety	Main results	Limitations
Takebayashi et al. (2010)	16 patients in remission from major depressive disorders, 16 healthy controls	DSM-IV	Not examined	No significant difference in plasma FGF2 levels was found between the MDD patients and the matched control subjects.	Limited sample size; Various medications
He et al. (2014)	28 pre- and post-treatment MDD patients (10 first episode and 18 recurrent episode), 30 healthy controls	DSM-IV, 24-item Hamilton Depression Rating Scale (HDRS-24)	Not examined	Serum FGF2 levels in MDD patients were significantly lower than those in healthy controls (*p* = 0.005), and the serum FGF2 levels decreased significantly but marginally following treatment for 8 weeks (*p* = 0.005).	Limited sample size; Different types of antidepressants
Kahl et al. (2009)	12 medication-free female patients with a major depressive episode in the context of borderline personality disorder (MDD/BPD), 12 healthy women	DSM-IV, German version of the Symptom Checklist (SCL-90-R), German version of the Beck Depression Inventory (BDI)	Not examined	Increased concentrations of FGF2 were found in MDD/BPD patients compared to the healthy group.	Limited sample size; No comparative group with current MDD and without BPD
Lu et al. (2013)	22 MDD patients with childhood trauma exposure (CTE), 21 MDD patients without CTE, and 22 healthy controls without CTE	DSM-IV, Zung’s Self-rating Depression Scale (SDS), 24-item Hamilton Depression Scale (HAMD)	Not examined	FGF2 was overexpressed in MDD patients with CTE only but not as much expressed in MDD patients without CTE.	Limited sample size; Biases caused by using questionnaires to assess histories of childhood trauma; Absence of a control group with CTE alone
Xu et al. (2017)	90 depressive patients (first episode and without drug treatment), 90 controls	Chinese classification of mental disorders- third Edition (CCMD-3), HDRS-24	Not examined	The patients presented significantly lower serum FGF22 levels, and the levels increased after 8 weeks of treatment.	The loose inclusion criteria (HDRS-24 = 8); No correlation analysis between FGF 22 levels and HDRS scores
Liu et al. (2017)	67 volunteers	Beck Depression Inventory (BDI), Self-Rating Anxiety Scale (SAS)	No correlation was found between FGF21 levels and SAS scores	A significant association was found between CSF FGF21 levels and BDI scores in male subjects, but not in female subjects.	No control group

*BDI, Beck Depression Inventory; BPD, borderline personality disorder; CCMD-3, Chinese Classification of Mental Disorders, Third Edition; CSF, Cerebrospinal fluid; CTE, childhood trauma exposure; DSM-IV, Diagnostic and Statistical Manual of Mental Disorders IV; FGF, fibroblast growth factor; HAMD, 24-item Hamilton Depression Scale; HDRS-24, 24-item Hamilton Depression Rating Scale; MDD, major depressive disorder; SAS, Self-Rating Anxiety Scale; SCL-90-R, Symptom Checklist-90-Revised; SDS, Self-rating depression scale*.

### Studies on Genetic Variation in FGF Genes

Many efforts have been made at the genetic level to explore the relations between FGFs and depression. Genetic variation of FGF2 can influence the therapeutic effect of antidepressant drugs. For example, several single-nucleotide polymorphisms (SNPs) in FGF2 gene were found to be associated with altered responsiveness to antidepressant treatment in individuals with MDD (Kato et al., 2009, 2015). On the contrary, no SNPs of FGFR2 gene was associated with depression (Wang et al., 2012). At the transcriptional level, the enrichment of FGF pathways is found both in depressed patients and rat models by gene expression analysis (Carboni et al., 2018).

### Role of FGFs in Depression

Fibroblast growth factor signaling has functional effects through different mechanisms. FGF2 increases the number or the survival of neurons in the hippocampus (Perez et al., 2009; Turner et al., 2011), and controls the development and size of the hippocampus (Ohkubo et al., 2004). Moreover, FGFR1 has been shown to directly interact with neurotransmitter receptors (adenosine 2A receptor and 5-HT1A receptor), and modulate neurochemistry (Flajolet et al., 2008; Borroto-Escuela et al., 2012). Interestingly, the FGF system may be able to compensate for the brain-derived neurotrophic factor (BDNF) system in the mesolimbic system of BDNF knockdown mice (Berton et al., 2006). Briefly, FGF ligands interact with FGF membrane receptors on the surface of neurons and glial cells or with voltage-gated sodium channels intracellularly. In addition, FGF receptors have partners such as neural cell adhesion molecules (NCAM) and 5-HT1A receptor (a G-protein coupled receptor). These events trigger a host of signaling pathways mentioned before (AKT, MAPK, PLCγ), and regulate neurogenesis, neuroplasticity or influence signal transduction. Furthermore, they regulate ongoing behavior including stress response, anxiety, motivated and affective behavior or episode of depression (Turner et al., 2012).

### FGFs in Other Mood Disorders

Besides depression, FGFs are associated with other mood disorders. The correlation between FGF2 and anxiety is most often elucidated. High-anxiety rats have lower levels of FGF2 mRNA in hippocampus compared with low-anxiety ones (Perez et al., 2009). The FGF2 knockdown in the hippocampus of rats by short-hairpin RNA silencing increases anxiety behavior (Eren-Kocak et al., 2011). Similarly, the FGF2 knockout was reported to increase hypothalamic-pituitary-adrenal axis (HPA) activity and anxiety behavior in mice (Salmaso et al., 2016). Moreover, peripheral FGF2 administration both in early-life and in adulthood of rats significantly reduces anxiety behavior (Perez et al., 2009; Turner et al., 2011). And the anxiolytic effect of fluoxetine must be mediated by FGF2 through the study of FGF2 knockout mice (Simard et al., 2018). In addition, FGF8 and FGF21 can also affect anxiety behavior, and they exert their influence in opposite directions. FGF8-deficient mice display increased anxiety-like behavior, while FGF21 administration in rats induces anxiogenic behavior (Brooks et al., 2014; Chiavaroli et al., 2017). As for bipolar disorder, serum levels of FGF2 are higher in patients with manic episode than those in healthy controls (Liu et al., 2014). FGF2 is related to bipolar disorder at the genetic level of humans (Xie et al., 2017). Similarly, FGFR2 is found to be associated with bipolar disorder through SNPs genotyping in humans (Wang et al., 2012).

## Discussion

Through reviewing existing studies, we found that the expression levels of some members of the FGF family were disordered in patients with depression, which indicates that a certain link exists between the FGF family and depression. But researches on the relation between FGF family and depression are not enough at present and the results of animal, postmortem and clinical studies have not been completely agreed.

Fibroblast growth factors and their roles in development, diseases, and therapies have been studied extensively. However, the study of FGFs in psychopathology, especially in depression, is an emerging field. To our best knowledge, it is the first review that focused on the neuromodulation effect of FGFs in depression. So far, there are limited studies showing the association between FGFs and depression, and the research methods are diverse and lack of homogeneity. Different experimental conditions, such as brain region, sample pH, and postmortem interval as we mentioned before, may lead to difference in the results of postmortem studies. Moreover, sample size, inclusion criteria, gender, therapeutic status of patients may play a role in the heterogeneity of results in clinical trials. In addition, previous studies only focused on the relations between FGF levels and depression, but seldom mentioned the deep connections between them and other depression-related disorders like anxiety. More attention should be paid on the signaling pathway and genetic variation of FGFs in depression, which may help to enrich the existing theories about the pathogenesis of depression.

## Author Contributions

ZD and SD wrote the manuscript. M-RZ searched the related literatures. M-MT planned and revised the manuscript. All authors read and approved the final version of the manuscript.

## Conflict of Interest Statement

The authors declare that the research was conducted in the absence of any commercial or financial relationships that could be construed as a potential conflict of interest.
